# Video Assisted Thoracoscopic Surgery Lung Volume Reduction for Chronic Obstructive Pulmonary Disease in Indonesia

**DOI:** 10.1186/1749-8090-10-S1-A110

**Published:** 2015-12-16

**Authors:** Karina Veronica Wilamarta, Tan Siauw Koan, Benjamin Yulianto Tanuwihardja, Nana Sunarya, Irwan Hananto

**Affiliations:** 1 Department of CardioThoracic Vascular Surgery, Husada and Medistra Hospital, Jakarta, 10001&12950, Indonesia; 2 Department of Radiology, Borromeus Hospital, Bandung, 40132, Indonesia; 3 Department of Pulmonology, Borromeus Hospital, Bandung, 40132, Indonesia; 4 Department of Pulmonology, Borromeus Hospital and Private Practice, 40132, Bandung, Indonesia; 5 Department of Pulmonology, Husada Hospital, Jakarta, 10001, Indonesia

## Background/Introduction

Chronic Obstructive Pulmonary Disease (COPD) in the future will be increased according to increasing air pollution by smoke from factories or vehicles and smoking habits. Asthma, chronic bronchitis, bronchiectasis, emphysema and others chronic and critical conditions require minimal invasive surgery.

## Aims/Objectives

Video Assisted Thoracoscopic Surgery (VATS) is a minimalist operation using a fiber optic cable that is connected to a video screen television for imaging objects and surgery will be done through several small incisions like keyhole of the chest cavity. VATS advantages compared to conventional surgery is the recovery time is shorter and very useful in situations whereas conventional thoracotomy surgery is not possible due to severe conditions. The drawback with this minimal incision exposes less due to obstructed ribs, but can be overcome with proper planning beforehand through a 3D reconstruction of CT Scans Thorax.

## Method

Case control study

## Results

Nine cases of COPD with pneumothorax complications between 44-84 years old performed VATS Lung Volume Reduction Surgery (LVRS). Comorbidities before surgery in 4 cases, such as Diabetes Mellitus, Coronary Arteriosclerosis Disease, emphysema subcutis, ulcer pepticum. Length of Stay (LOS) 5-40 days, morbidity infection in 3 cases, 1 case of gastrointestinal bleeding, prolonged pneumothorax 2 cases, 1 recurrent pneumothorax and no mortality. Functional testing of lung vital capacity about 21.5% with mean forced expiratory volume of 20%. Spirometry evaluations during the year showed an increase of 10-20%, but the functional of daily activities showed improvement and free from oxygen dependency. Four patients developed congestion right heart began three months after the respiratory rehabilitation with one person showed moderate pulmonary hypertension. 4-year follow-up to the dismissal of contamination to cigarette smoke showed significant changes in lung function of ventilation and perfusion test in Nuclear Medicine

## Discussion/Conclusion

VATS LVRS showed good outcome for selected COPD.

